# Substance use and disordered eating risk among college students with obsessive-compulsive conditions

**DOI:** 10.1371/journal.pone.0316349

**Published:** 2025-01-02

**Authors:** Wura Jacobs, Angela DeLeon, Alane Bristow, Patrick Quinn, Alyssa Lederer

**Affiliations:** 1 Department of Applied Health Science, School of Public Health, Indiana University, Bloomington, Indiana, United States of America; 2 Preventive Medicine, Keck School of Medicine, University of Southern California, Los Angeles, California, United States of America; University of Kansas School of Medicine Wichita, UNITED STATES OF AMERICA

## Abstract

**Purpose:**

College students are at higher risk for problematic substance use and disordered eating. Few studies have examined the comorbid risks associated with OCD despite the increased prevalence of OCD among young adults. This study examined substance use and disordered eating risk associated with OCD conditions among college students and how this association may vary by sex/gender.

**Methods:**

Data were from 92,757 undergraduate students aged 18–24 enrolled in 216 colleges between Fall 2021 and Fall 2022, from the American College Health Association-National College Health Assessment III. Regression models were used to estimate alcohol, cannabis, tobacco, and disordered eating risk among those with OCD related conditions compared to those without conditions, overall and by sex/gender, while adjusting for covariates and school clustering.

**Results:**

Students with OCD conditions displayed a higher prevalence of substance use and disordered eating risks. In adjusted models, OCD conditions were associated with increased odds of moderate/high tobacco (aOR = 1.12, 95% CI 1.05, 1.21), cannabis (aOR = 1.11, 95% CI 1.04, 1.18), alcohol (aOR = 1.14, 95% CI 1.05, 1.24) and disordered eating risk (aOR = 2.28, 95% CI 2.13, 2.43). Analyses stratified by gender revealed cis-female students with OCD conditions were at increased risk for moderate/high risk alcohol (aOR = 1.18, 95% CI 1.08, 1.29), tobacco (aOR = 1.12, 95% CI 1.03, 1.22), cannabis (aOR = 1.13, 95% CI 1.06, 1.23) and disordered eating (aOR = 2.30, 9%% CI 2.14, 2.47). Among TGNC students, OCD conditions were associated with increased risk for moderate/high tobacco risk (aOR = 1.24, 95% CI 1.05, 1.48) and disordered eating (aOR = 2.14, 95% CI 1.85, 2.47). OCD conditions was only associated with disordered eating among male students (aOR = 2.34, 95% CI 1.93, 2.83).

**Discussion:**

Young adult college students with OCD conditions exhibit a higher prevalence of medium/high risk alcohol, tobacco, and cannabis use and disordered eating compared to their counterparts without such conditions, even after adjusting for stress, depression, and anxiety.

## Introduction

Young adults represent a significant proportion of new obsessive-compulsive disorder (OCD) diagnoses [1]. Although previously thought to be a rare condition, OCD is now recognized as a prevalent mental health condition and substantial contributor to the global burden of disease [2, 3]. The worldwide prevalence of OCD is estimated at 1–3% of the population [4, 5]. Within the U.S., one study estimated lifetime and 12-month prevalence of OCD at 2.3% and 1.2%, respectively [6]. Moreover, the prevalence of those meeting the Diagnostic and Statistical Manual of Mental Disorders, Fifth Edition (DSM-5) criteria for OCD does not capture the substantial subsyndromal variation in OCD symptoms, with approximately 28.2%, 12.9%, and 6.2% of U.S. adults reported having ≥1, ≥2, or ≥3 OCD symptoms for ≥2 weeks in their lifetime, respectively [7].

OCD exemplifies the class of conditions categorized as “obsessive-compulsive and related disorders” (e.g., trichotillomania, hoarding) in the DSM-5 [3]. Obsessions are perseverative thoughts, images, or urges that are unwanted, intrusive, and extremely anxiety-provoking [4]. Compulsions are behaviors or mental acts that are repetitive and ritualistic, performed with the goal of transiently alleviating the anxiety or distress associated with obsessive thoughts [4, 8].

There is extensive evidence that OCD is often comorbid with mood, anxiety, and other psychotic disorders [1]. Eating disorders (EDs) have also long been known to be comorbid with OCD with estimates of co-occurrence ranging from 35–44% in some reports [9]. While OCD and substance use disorder (SUD) are typically considered clinically and nosologically separate, they exhibit common features in their phenomenology, which is often characterized by compulsive activities [10]. Recent evidence shows that lifetime co-occurrence of substance dependence is higher among individuals with OCD [11]. However, these studies are mostly among clinic- or community-based general adult population and do not focus on the population most at-risk for onset of OCD—young adults.

Beyond increasing anxiety and depression rates among young adults, recent trends also show an increase in diagnoses and treatment for other mental health concerns such as OCD (from 1.9% in 2009 to 2.4% in 2015) among college students [12]. Despite evidence showing an overall decline in mental health, increasing OCD prevalence among young adult college students, and the comorbid risks associated with OCD broadly, there remains scant evidence specific to co-occurrence of OCD with alcohol, tobacco, cannabis, and disordered eating risk among young adults [7, 11, 13, 14]. With the increasing prevalence of substance use among college students with mental health concerns [15], understanding comorbid disorders is important because comorbid psychiatric conditions and risk behaviors have an impact on condition prognosis, symptom exacerbation, and efficacy of treatments [16].

The current study aims to fill this gap by providing empirical evidence to better understand the substance use and disordered eating risks associated with OCD conditions among college students. We additionally explored variation in these associations across sex/gender given differences in the prevalence of OCD and substance use by sex/gender.

## Methods

### Study population

Data were from the American College Health Association-National College Health Assessment III (ACHA-NCHA III), a web-based, national survey of U.S. college student health administered each semester [17]. Sponsored by the American College Health Association (ACHA), the ACHA-NCHA III is the third major iteration of the ACHA-NCHA and was first implemented in Fall 2019. Due to the disruption and unusual problems caused by the COVID-19 pandemic, the present study uses ACHA-NCHA III data collected from students enrolled in 216 colleges and universities between Fall 2021 and Fall 2022. Data pooled from these three periods were further restricted to undergraduate students of traditional age (18–24 years) [18]. To be eligible for inclusion in the ACHA-NCHA III national dataset, participating institutions had to sample a census or random sample of students >18 years. Participants provided written informed consent before completing the survey, and a university Institutional Review Board approved the secondary analysis of the ACHA-NCHA data for this study (IRB# 18760). The ACHA-NCHA III data used in this study is available upon request made to the American College Health Association through their website (https://www.acha.org/ncha/data-results/data-access-published-literature/) or email (ncha@acha.org).

## Measures

### Primary outcome variables

#### Substance use risk

Risk of use of three substances of interest were examined in this study: alcohol, tobacco, and cannabis. ACHA-NCHA III uses the Alcohol, Smoking and Substance Involvement Screening Test (ASSIST) to measure substance specific involvement risk among college students [19]. ASSIST is an 8-item questionnaire that asks about lifetime use of substances and use of substances and associated problems over the last 3 months. Responses to these questions are used to calculate students’ ASSIST risk scores ranging from 0–39. Scores are used to categorize students into low risk (0–3), moderate risk (4–26), or high risk (27–39) for tobacco and cannabis use. For alcohol, categories were 0–10 (low risk), 11–26 (moderate risk), 27–39 (high risk). Students who indicated no lifetime use of any of the three substances were coded as low risk. Given the interest of the study in examining determinants of risk, participants were collapsed into two groups: medium/high risk (≥27) and not medium/high risk (<27).

#### Eating disorder

Two questions were used to assess eating disorder (ED) behavior or diagnoses among students. One question asked participants if “within the last 12 months, an eating disorder /problem affected your academic performance?” A second question asked if they had “ever been diagnosed by a healthcare or mental health professional with eating disorders (for example: anorexia nervosa, bulimia nervosa, binge eating.” Responses to these two questions were combined into a categorical variable where 0 = no eating disorder behavior/diagnoses and 1 = experience of eating disorder behavior/diagnoses.

### Primary predictor variable

#### Obsessive-compulsive and related conditions

Indication of diagnosis was obtained with a question (yes or no) asking whether students have “ever been diagnosed by a healthcare or mental health professional as having obsessive-compulsive and related conditions (for example: OCD, Body Dysmorphia, Hoarding, Trichotillomania, other body-focused repetitive behavior disorders. Responses were coded such that 0 = no diagnosis and 1 = positive diagnosis for OCD related conditions.

### Study covariates

#### Sex/gender

ACHA used responses to three questions on sex at birth, gender identity, and whether students identify as transgender to create a sex/gender variable. When a student’s gender identity was consistent with their sex at birth and they selected no for transgender, sex/gender was coded as cisgender woman or cisgender man. Students were categorized as transgender/gender non-conforming (TGNC) if they selected “yes” for transgender, “intersex” for sex at birth, or their sex at birth was not consistent with their gender identity. Students who skipped any of the three questions used to compute the variable were coded as missing. A full description of the methodology ACHA employed in computing the sex/gender variable is published elsewhere [20].

#### Other covariates

Given the established relationship between stress, mental health factors, substance use, and OCD conditions [10, 21], binary indications of past 12-month stress (moderate/high vs. no/low), previous diagnosis of anxiety (yes/no), and previous diagnosis of depression (yes/no) were included as covariates. Additional covariates included self-reported demographic information: age (in years); race/ethnicity (Non-Hispanic White [White], Non-Hispanic Black [Black], Hispanic/Latinx, Non-Hispanic Asian/Asian American [Asian], Non-Hispanic American Indian/Alaskan Native/Native Hawaiian/Pacific Islander [NHOPI], Middle Eastern/other Arab [MENA], Biracial/Multicultural (if more than one race/ethnicity selected), Other); parent highest education level (ranging from did not finish high school to doctoral/professional degree); and survey year (Fall 2021, Spring 2022, Fall 2022).

### Statistical analysis

This study employed a cross-sectional design. Descriptive statistics were used to summarize the data. Bivariate associations between the primary outcomes (alcohol, tobacco and cannabis risk, eating disorder) and OCD conditions were evaluated using univariate logistic regression analyses. To adjust for student-institution clusters, obtain more efficient parameter estimates, and better standard errors, multivariable regression models were constructed, applying generalized estimating equations (GEE) method. For each outcome, a GEE model with an exchangeable correlation structure and robust variance estimator was used to estimate the alcohol, cannabis, tobacco, and disordered eating risk among those with OCD conditions compared to those without. The models adjusted for study covariates including sex/gender. To examine whether risks differed by students’ sex/gender, we conducted stratified multivariable analyses for each sex/gender. The models stratified by sex/gender included the same set of covariates but excluded sex/gender. Additionally, predicted probabilities (average marginal effects) of medium/high substance use and eating disorder risks by sex/gender were estimated based on the adjusted regression models [22]. Multicollinearity was assessed with the variance inflation factor (VIF) and all VIF values (≤1.90) were below the recommended threshold (≥2.50) indicating no multicollinearity concerns [23]. Missing data on the study’s primary predictor, OCD condition, was 0.8% while missingness for other study variables ranged from 0.2% to 3.5%. Given the small amount of data missingness relative to sample size, we employed listwise deletion [24, 25]. All analyses were completed using Stata version 18.0 software (StataCorp, LLC).

## Results

As Table 1 shows, 92,757 undergraduate students between the ages of 18 and 24 (mean age 19.88 years (SD = 1.45)) were included in this study. The majority of respondents were White (62%) and identified as cisgender female (63%), and about 6% of students in the sample identified as TGNC. About 6% of respondents indicated having OCD conditions. Among the study sample, 12%, 16%, and 19% of students were classified as having medium/high alcohol, tobacco, and cannabis risk respectively (Table 2). Disordered eating behaviors were reported among 19% of students in the sample. Across sex/gender, 23%, 8%, and 36% of cisgender female, cisgender male, and TGNC students reported eating disorders. Among students with OCD conditions, prevalence of medium/high substance use and disordered eating risks were significantly higher (Table 2). In the bivariate analyses examining association of OCD conditions with medium/high substance use risk, results showed students with OCD conditions to be at significantly higher likelihood (p<0.001) to be classified as having medium/high alcohol, tobacco, and cannabis use risk (Table 2). Students with OCD conditions also had higher odds of reporting disordered eating (p<0.001).

**Table 1:** Descriptive characteristics of study sample and by OCD exposure (N = 92,757).

Variables	Overall	OCD Exposure	
		Yes (n = 5,469)	No (n = 86,579)
Age (years), mean (SD)	19.88 (1.45)	19.99 (1.48)	19.87 (1.45)
Sex (%)			
Male	27,969 (30.15)	721 (13.18)	27,025 (31.21)
Female	58,314 (62.87)	3,847 (70.34)	54,047 (62.43)
Transgender/gender non-conforming	5,710 (6.16)	810 (14.81)	4,861 (5.61)
Race/ethnicity (%)			
White	57,252 (61.72)	4,099 (74.95)	52,816 (61.00)
Black	4,375 (4.72)	91 (1.66)	4,234 (4.89)
Asian	10,260 (11.06)	221 (4.04)	9,924 (11.46)
Latinx	7,794 (8.40)	206 (3.77)	7,521 (8.69)
NHOPI	545 (0.59)	29 (0.53)	513 (0.59)
MENA	769 (0.83)	36 (0.66)	720 (0.83)
Bi/Multiracial	11,039 (11.90)	745 (13.62)	10,221 (11.81)
Other	428 (0.46)	26 (0.48)	26 (0.48)
Generalized Anxiety (%)			
Yes	29,573 (31.88)	4,828 (88.28)	24,619 (28.44)
No	62,606 (67.49)	633 (11.57)	61,836 (71.42)
Depression (%)			
Yes	22,589 (24.35)	3,878 (70.91)	18,615 (21.50)
No	69,515 (74.94)	1,580 (28.89)	67,804 (78.31)
Stress (%)			
Moderate/High	73,460 (79.20)	4,990 (91.24)	67,950 (78.48)
No/Low	19,154 (20.65)	477 (8.72)	18,550 (21.43)
Parents education (%)			
Don’t know	1,101 (1.19)	50 (0.91)	1,045 (1.21)
Did not finish high school	2,636 (2.84)	80 (1.46)	2,535 (2.93)
High school or GED	13,274 (14.31)	591 (10.81)	12,575 (14.52)
Some college	7,217 (7.78)	424 (7.75)	6,753 (7.80)
Associate degree	7,020 (7.57)	389 (7.11)	6,592 (7.61)
Bachelor’s degree	26,928 (29.03)	1,546 (28.27)	25,238 (29.15)
Master’s degree	23,377 (25.20)	1,585 (28.98)	21,664 (25.02)
Doctoral degree	10,947 (11.80)	799 (14.61)	10,055 (11.61)
Study semester (%)			
Fall 2021	21,759 (23.46)	1,033 (18.89)	20,512 (23.69)
Spring 2022	49,426 (53.29)	3,043 (55.64)	46,068 (53.21)
Fall 2022	21,572 (23.26)	1,393 (25.47)	19,999 (23.10)

Note: NHOPI = Non-Hispanic American Indian/Alaskan Native/Native Hawaiian/Pacific Islander. MENA = Middle Eastern/other Arab

**Table 2:** Bivariate associations between OCD related conditions and primary outcomes among overall study population (N = 92,757).

Variable	Overall	OCD related conditions	OR, 95% CI
	%	%	(p value)*
ASSIST^†^ Scores			
Alcohol risk (%)			1.58, 1.46–1.71 (<0.001)
Medium/high	12.20	17.74	
Not medium/high	84.34	78.90	
Tobacco risk (%)			1.64, 1.53–1.76 (<0.001)
Medium/high	16.07	24.12	
Not medium/high	82.84	74.99	
Cannabis risk (%)			1.81, 1.71–1.93 (<0.001)
Medium/high	19.24	30.52	
Not medium/high	78.88	67.22	
Eating disorder (%)			
Yes	19.19	46.22	4.05, 3.80–4.32 (<0.001)
No	80.51	53.65	

*Significance of association of OCD related conditions with primary outcomes analyzed with univariate binomial logit analysis using GEE methods adjusted for clustering by school.

^†^ASSIST = Alcohol, Smoking and Substance Specific Involvement Test

### Association between OCD conditions and substance use risk

Table 3 shows the adjusted associations between OCD conditions and substance use and disordered eating risk in the full sample and stratified by sex/gender. Overall, adjusting for covariates including stress, depression, and anxiety, having OCD conditions were associated with greater odds of medium/high tobacco (adjusted odds ratio [aOR] = 1.12, 95% confidence interval [95% CI] 1.05, 1.21), cannabis (aOR = 1.11, 95% CI 1.04, 1.18), and alcohol (aOR = 1.14, 95% CI 1.05, 1.24) risk. In the models stratified by sex/gender, for cisgender female students, OCD condition was associated with greater odds of medium/high tobacco (aOR = 1.12, 95% CI 1.03, 1.22), cannabis (aOR = 1.13, 95% CI 1.06, 1.23), and alcohol (aOR = 1.18, 95% CI 1.08, 1.29) risk. There were no statistically significant associations between OCD conditions and substance use risk among cisgender male students in the study. However, among TGNC students, OCD conditions were only statistically significantly associated with higher odds of medium/high tobacco risk (aOR = 1.24, 95% CI 1.05, 1.48).

**Table 3:** Overall and gender-stratified generalized estimating equation (GEE) models with medium/high risk of tobacco, cannabis, and eating disorder as the dependent variables.

	Tobacco	Cannabis Risk	Alcohol Risk	Eating disorder
	aOR [95% CI]	aOR [95% CI]	aOR [95% CI]	aOR [95% CI]
Full sample (N = 92,757)				
OCD conditions	1.12**	1.11**	1.14**	2.28***
	[1.05, 1.21]	[1.04, 1.18]	[1.05, 1.24]	[2.13, 2.43]
Stress	1.33***	1.41***	1.54**	1.94***
	[1.27, 1.39]	[1.35, 1.48]	[1.45, 1.63]	[1.83, 2.05]
Depression	1.82 ***	2.05***	1.64***	2.15***
	[1.72, 1.92]	[1.94, 2.17]	[1.55, 1.75]	[2.03, 2.26]
Anxiety	1.12***	1.17***	1.01	1.16***
	[1.91, 1.78]	[1.11, 1.23]	[0.95, 1.07]	[1.10, 1.22]
Cis-Females (n = 58,314)				
OCD conditions	1.12**	1.13**	1.18***	2.30***
	[1.03, 1.22]	[1.06, 1.23]	[1.08, 1.29]	[2.14, 2.47]
Stress	1.31***	1.37***	1.43***	1.84***
	[1.22, 1.40]	[1.28, 1.46]	[1.33, 1.54]	[1.72, 1.97]
Depression	1.90 ***	2.10***	1.64***	2.19***
	[1.78, 2.02]	[1.96, 2.26]	[1.53, 1.76]	[2.06, 2.32]
Anxiety	1.13***	1.17***	1.04	1.14***
	[1.06, 1.21]	[1.10, 1.25]	[0.97, 1.12]	[1.08, 1.21]
Cis-Males (n = 27,969)				
OCD conditions	0.99	0.97	1.07	2.34***
	[0.82, 1.21]	[0.81, 1.17]	[0.86, 1.33]	[1.93, 2.83]
Stress	1.35***	1.47 ***	1.65***	2.35***
	[1.26, 1.46]	[1.36, 1.58]	[1.49, 1.83]	[2.10, 2.64]
Depression	1.58***	1.85***	1.63***	2.14***
	[1.40, 1.78]	[1.67, 2.05]	[1.44, 1.85]	[1.84, 2.49]
Anxiety	1.17**	1.24***	0.97	1.22**
	[1.05, 1.31]	[1.13, 1.36]	[0.87, 1.09]	[1.05, 4.41]
TGNC (n = 5,710)				
OCD conditions	1.24 *	1.12	1.03	2.14***
	[1.05, 1.48]	[0.95, 1.33]	[0.81, 1.32]	[1.85, 2.47]
Stress	1.38 **	1.48***	1.79**	1.76***
	[1.08, 1.76]	[1.23, 1.79]	[1.28, 2.52]	[1.45, 2.13]
Depression	1.97***	2.18***	1.75***	1.90***
	[1.57, 2.46]	[1.84, 2.57]	[1.34, 2.28]	[1.64, 2.21]
Anxiety	0.93	1.06	0.87	1.16
	[0.75, 1.16]	[0.89, 1.27]	[0.69, 1.09]	[0.99, 1.37]

Note: Models also adjust for demographic characteristics including age, parent highest education level and race/ethnicity and semester of data collection. aOR = adjusted odds ratio

*p < .05.

**p < .01.

***p < .001 TGNC = Transgender/gender non-conforming

### Association between OCD conditions and disordered eating behaviors

Similar to findings for substance use risk, OCD condition was associated with greater risk of disordered eating (aOR = 2.28, 95% CI 2.13, 2.43) in the overall sample. In the models stratified by sex/gender, OCD condition was associated with greater risk of disordered eating among cisgender females (aOR = 2.30, 95% CI 2.14, 2.47), cisgender males, (aOR = 2.34, 95% CI 1.93, 2.83), and TGNC students (aOR = 2.14, 95% CI 1.85, 2.47).

### Average marginal effects of OCD conditions on substance use risk and disordered eating behaviors by sex/gender

**Fig 1:**
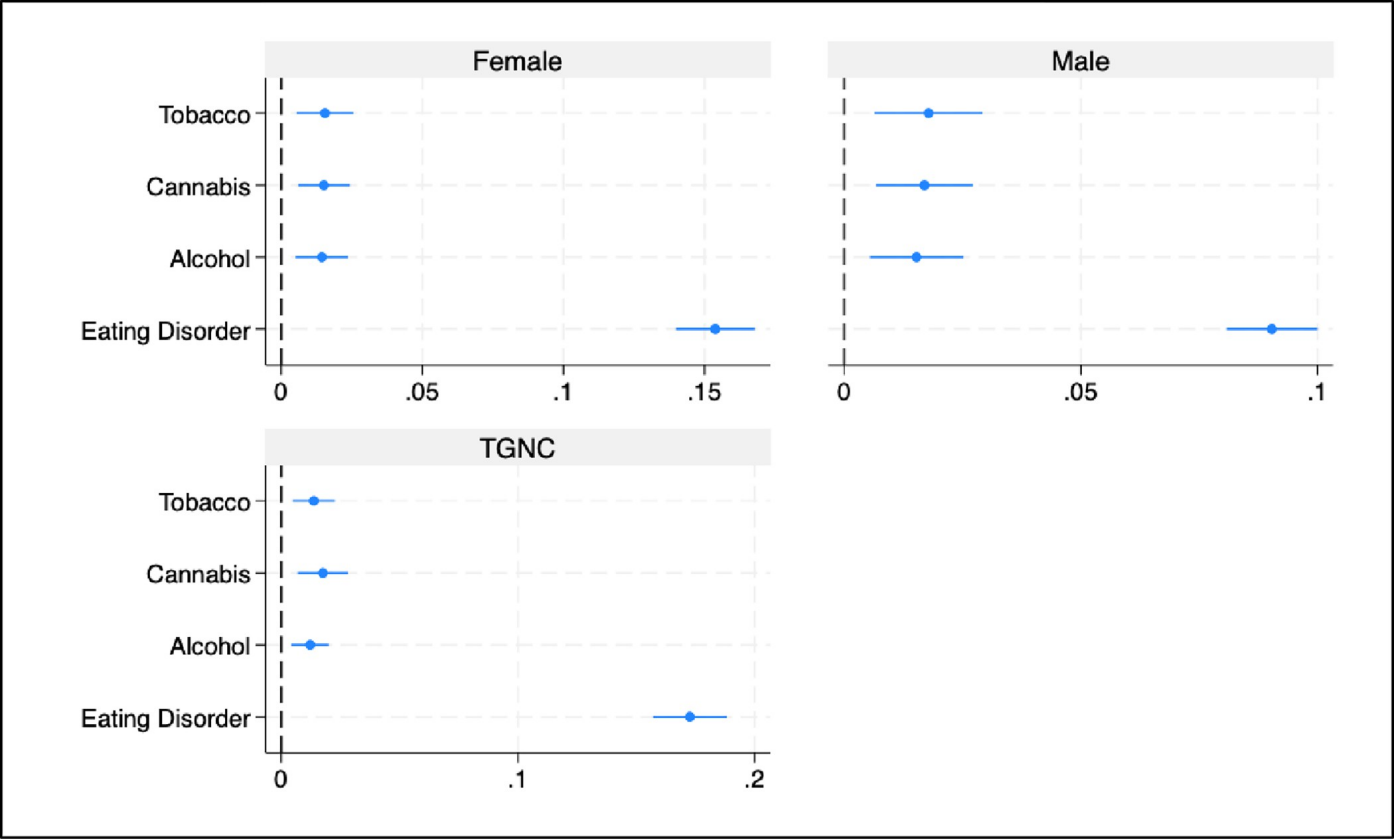
Predicted probabilities of medium/high tobacco, cannabis, alcohol and eating disorder risk by sex/gender. Note. TGNC: Transgender/gender non-conforming.

As shown in Fig 1, having OCD conditions was associated with a statistically significant increase in the predicted probability of tobacco (0.02, 0.02, 0.01; p = 0.001), cannabis (0.02, p = 0.001), and alcohol (0.02, 0.02, 0.01; p<0.002) risk among cisgender female, cisgender male and TGNC students, respectively. For eating disorder, predicted probabilities were 0.15, 0.09, and 0.17 (p<0.001) for cisgender female, male and TGNC students, respectively (Fig 1).

## Discussion

To our knowledge, this is the first study to examine substance use and disordered eating risks associated with OCD conditions among young adult college students in the U.S. as well as how these associations may vary among cisgender male, cisgender female, and TGNC students. Our findings show that overall, college students with OCD conditions showed higher prevalence of medium/high alcohol, tobacco, and cannabis involvement and disordered eating risk compared to their counterparts without OCD conditions. Even when accounting for stress, depression, and anxiety, having an OCD condition was associated with greater likelihood of medium/high alcohol, tobacco, cannabis, and disordered eating risk; however, the associations varied by sex/gender.

Previous studies have reported that due to the similarities in their phenomenology, and the increased likelihood of individuals with OCD to turn to substances to self-medicate and alleviate distress caused by obsessive thoughts and compulsive behaviors, there is a high level of comorbidity between OCD and substance use risk and disorder [10, 11, 26]. In line with these studies, we found that OCD conditions were associated with elevated odds of medium/high alcohol, tobacco, and cannabis use risk. Although most of the previous studies were conducted among a general adult population and in international samples [26], our study extends current knowledge by demonstrating the association of OCD conditions with medium/high risk alcohol, tobacco, and cannabis use in a U.S. sample among a population subgroup (young adult college students, 18–24 years) that comprises a significant fraction of the U.S. population (38%) and represents a large proportion of new OCD diagnoses [27–29].

OCD and substance misuse share similar neurological and genetic influences [26]. However, there is heterogeneity in their presentation due to the interaction between genetic and biological risk factors and a range of environmental influences [30, 31]. The elevated odds of alcohol, tobacco, and cannabis use among students with OCD may reflect these shared influences, as well as the unique risks posed by the college environment. College campuses are unique environments that foster high-risk substance use (e.g., polysubstance use) through factors like Greek life affiliations [32], academic stress [33], and social norms that encourage substance use [34]. Given that OCD is a multifactorial condition influenced by environmental contexts, students with OCD may be particularly vulnerable to substance use disorders in such settings. Our findings underscore the importance of incorporating regular substance use screenings into the clinical care of college students with OCD. Furthermore, tailoring support systems to address both the anxiety and compulsions characteristic of OCD and the environmental triggers unique to college life is essential for promoting the well-being of these students.

In line with studies indicating that sex/gender significantly influences the expression and impact of OCD [1, 5, 35, 36], this study demonstrates that the risks and comorbidities associated with OCD among college students vary by sex/gender. Even after accounting for other stress and mental health symptoms, cisgender females with OCD had a higher risk of comorbidity related to medium/high alcohol, tobacco, and cannabis use as well as disordered eating. TGNC students with OCD conditions were at risk for both medium/high tobacco use and disordered eating while cisgender males with OCD conditions showed an elevated risk only for disordered eating. Our results fill a crucial gap and advance the literature by providing sex/gender-stratified evidence from a U.S.-based population subgroup (traditional age college students) that is not currently represented in the literature.

Earlier studies have reported mixed findings regarding gender, OCD symptomatology, and comorbid conditions [5, 35, 37]. For example, one study found no difference in the prevalence of lifetime comorbidities between males and females [37], another reported an elevated risk of substance use disorder risk among males [1], and yet another found elevated risk of social phobia and eating disorders among females [5]. While no studies have specifically addressed OCD comorbidities among TGNC individuals, the mixed findings for cisgender males and females may stem from differences in study populations (children vs. adults vs. college students; community vs. clinical samples) or the geographical context (i.e. different countries) [37]. Another possible explanation for this variability could be sex/gender differences in symptom onset and presentation [37]. For example, males with OCD conditions are more likely to exhibit social anxiety disorder and tic disorders, while females are more prone to eating and impulse control disorders [37]. Additionally, research has shown that cisgender women and TGNC individuals often experience heightened psychological distress related to social expectations discrimination, and internalized stigma which may exacerbate obsessive-compulsive behaviors and increase their risk of medium/high substance use and disordered eating [38]. The observed sex/gender variability in risks and comorbidities associated with OCD among college students may also stem from cultural influences and socioenvironmental factors interacting with biological predispositions that shape risk behaviors [35]. These findings emphasize that while all college students with OCD conditions are an at-risk group, cisgender female students face the highest risks and should be prioritized for tailored interventions and programs to support healthy symptom management and effective treatment planning. Moreover, the results highlight the significant sex/gender differences in the complex interplay between psychosocial stressors, OCD symptomatology, and maladaptive coping behaviors, underscoring the need for nuanced approaches to address these challenges.

Among non-college populations, there is evidence showing a high comorbidity between eating disorders (EDs) and OCD, attributed to their shared genetic and psychological features [16]. It is therefore not surprising that, among young adult college students with OCD conditions, EDs emerged as the only consistent comorbid risk. However, unlike previous studies that identified cisgender females with OCD as the primary groups at elevated risk for EDs [5, 39], our findings revealed that among college students, all sexes/genders are at greater risk, with predicted risk probabilities highest among TGNC students. This highlights the unique comorbid risks associated with OCD conditions among college students.

First, it is important to note that the disordered eating variable examined in this study encompassed multiple disorders including anorexia nervosa, bulimia nervosa, and binge eating. College students are particularly vulnerable to body dissatisfaction, weight concerns, and appearance-related stress, which are amplified by the college environment and life stage. Disordered eating behaviors, such as restrictive eating, binge eating, and purging, are prevalent in this population and are further exacerbated by stress related to academic performance, relationship challenges, and the transition to college life [40, 41]. Both OCD and EDs often involve ritualistic behaviors as well as intrusive thoughts including those related to body image, weight, and food. The heightened risk of EDs among students with OCD conditions is especially concerning because college students are at a developmental stage marked by susceptibility to mental disorders [42], reluctance to seek professional help [43], and the formation of long-term behavioral patterns that influence their quality of life, chronic disease risks, and overall health. The pervasive risk of EDs among cisgender males, cisgender females, and TGNC students with OCD conditions underscores the need for comprehensive mental health screening and tailored support systems within college settings.

In summary, our findings show that college students with OCD conditions—a complex, multifaceted disorder with comorbidity risks varying by sex/gender—are an at-risk group for engaging in various risk behaviors. However, these results should be interpreted within the context of our study limitations. First, while this study employed one of the best national datasets available on college students’ health which includes data on diverse racial and gender identities, it is not nationally representative of college students in the U.S. Additionally, the cross-sectional nature of the data preclude the ability to establish causality and the limited confounder adjustment (e.g., no data on other mental health issues such as PTSD, sleep disorders) could influence study outcomes. It is also important to note that OCD and ED measures examined in the study were based on self-reported, single-item measures regarding clinically diagnosed conditions. Although these measures are useful for screening purposes, they do not allow us to disentangle specific eating disorder type (e.g., anorexia nervosa, binge eating) or obsessive compulsive and related disorders (e.g., perseverative thoughts, repetitive behaviors, body dysmorphia, hoarding, trichotillomania) that may have been the primary drivers of reported associations. Furthermore, although the sample included TGNC students, the relatively small size of this subgroup may have limited the study’s power to detect additional associations. Despite these limitations, this study’s contributions to the extant literature fill an important knowledge gap on the risks associated with OCD conditions among young adults in the U.S.

This study represents a pioneering investigation of the complex interplay between OCD conditions, substance use, and disordered eating risks among U.S. young adult college students. Our findings reveal that students with OCD conditions have a higher prevalence of medium/high risk alcohol, tobacco, and cannabis use and disordered eating compared to their counterparts without such conditions, even after adjusting for stress, depression, and anxiety. Although cisgender male, cisgender female, and TGNC students with OCD conditions were at greater risk for eating disorders, TGNC students exhibited the highest predicted risk probability while cisgender females had the greatest comorbidity risks. The study underscores the unique challenges posed by the college environment and life-stage—where substance use and disordered eating behaviors are amplified—that may predispose young adults with OCD to developing substance use and eating disorders. The heightened risk for eating disorders emphasizes the significance of comprehensive mental health screening and support on college campuses. Our findings contribute valuable insights into the underexplored intersection of OCD, substance use, and disordered eating risks among college students, highlighting the imperative for future research to unravel the intricate sex/gender differences in the biopsychosocial mechanisms underlying these associations.
