# Correction: Substance use and disordered eating risk among college students with obsessive-compulsive conditions

**DOI:** 10.1371/journal.pone.0321378

**Published:** 2025-03-28

**Authors:** Wura Jacobs, Angela De Leon, Alane Bristow, Patrick Quinn, Alyssa Lederer

The second author’s name is spelled incorrectly. The correct name is: Angela De Leon. The correct citation is: Jacobs W, De Leon A, Bristow A, Quinn P, Lederer A (2025) Substance use and disordered eating risk among college students with obsessive-compulsive conditions. PLoS ONE 20(1): e0316349. https://doi.org/10.1371/journal.pone.0316349

## References

[1] Jacobs W, De Leon A, Bristow A, Quinn P, Lederer A. Substance use and disordered eating risk among college students with obsessive-compulsive conditions. PLoS One. 2025;20(1): e0316349. doi: 10.1371/journal.pone.0316349 39746071 PMC11694961

